# Abundance, Diversity, and Function of Soil Microorganisms in Temperate Alley-Cropping Agroforestry Systems: A Review

**DOI:** 10.3390/microorganisms10030616

**Published:** 2022-03-15

**Authors:** Lukas Beule, Anna Vaupel, Virna Estefania Moran-Rodas

**Affiliations:** 1 Julius Kühn Institute (JKI)—Federal Research Centre for Cultivated Plants, Institute for Ecological Chemistry, Plant Analysis and Stored Product Protection, 14195 Berlin, Germany; lukas.beule@julius-kuehn.de (L.B.); anna.vaupel@julius-kuehn.de (A.V.); 2 Department of Crop Sciences, Division of Agricultural Entomology, University of Goettingen, Grisebachstraße 6, 37077 Goettingen, Germany

**Keywords:** agroforestry, alley cropping, tree-based intercropping, temperate zone, soil microorganisms, soil microbial activity, soil microbial diversity

## Abstract

Modern temperate alley-cropping systems combine rows of trees with rows of crops (agroforestry), which allows for diverse interspecific interactions such as the complementary and competitive use of resources. The complementary use of resources between trees and crops is considered the main advantage of these multifunctional land use systems over cropland monocultures. Moreover, several studies demonstrated that agroforestry systems are environmentally more sustainable than cropland monocultures. Over two decades of research on soil microorganisms in temperate alley-cropping systems are characterized by a variety of different methodological approaches and study designs to investigate the impact of agroforestry on the soil microbiome. Here, we review the available literature on the abundance, diversity, and functionality of soil microorganisms in temperate alley-cropping systems. Further, we identify current knowledge gaps as well as important experimental factors to consider in future studies. Overall, we found that temperate alley-cropping systems increase soil microbial abundance, diversity, and functions as compared to cropland monocultures, which is expected to contribute to enhanced biological soil fertility in these systems.

## 1. Introduction

Agroforestry systems are agricultural systems that combine trees or other woody plants with crops or livestock. Several different systems fall under the umbrella of agroforestry. Temperate agroforestry systems include, among others, alley-cropping systems, shelterbelts, orchard meadows, and forest pastures. In the temperate zone, alley-cropping systems are gaining popularity as they can maintain or even increase production while being environmentally more sustainable than cropland monocultures [1,2]. Modern alley-cropping systems in the temperate zone combine fast-growing trees (e.g., poplar, willow) with annual crops (e.g., wheat, maize, and oilseed rape). The tree and crop components of these systems are often arranged in alternating alleys with North–South orientation to minimize shading. The spatial proximity of the trees and crops allows for a variety of interspecific interactions in these systems such as competition for resources as well as complementary resource use [3]. Over 25 years ago, Cannell et al. [4] argued that yield benefits of agroforestry systems only occur if resources are used in a complementary manner by the trees and crops—a hypothesis dubbed the “central hypothesis of agroforestry”. Twenty-five years later, a large body of literature has shown that the benefits of complementary resource use can outweigh the disadvantages of resource competition in agroforestry systems.

The most prominent example of complementary resource use is the altered soil–nutrient (re)cycling in agroforestry systems. In 2004, Allen and coworkers [5] found evidence that trees in temperate agroforestry systems can reduce nutrient leaching by taking up leachable nutrients that were not utilized by the crops. These otherwise lost nutrients are incorporated into the biomass of the trees and will partly reenter the soil through tree litter. This recycling of nutrients has been described as ‘nutrient pumping’ [6]. The supply of nutrients through tree litter is likely to account for increased soil fertility close to the trees [7]. In addition to their function as nutrient pumps, trees effectively reduce wind speed [8] and, thus, can contribute to reduced soil erosion. Furthermore, trees can also decrease arthropod pests, increase the abundance of natural enemies, e.g., [9], and enhance pollination services, e.g., [10]. Unsurprisingly, the integration of trees through agroforestry also increases biodiversity, e.g., [11], and C sequestration of agroecosystems, e.g., [12]. In addition to environmental benefits, the woody biomass of the trees serves as an additional product for farmers [13]. This economic diversification may help to compensate for fluctuations in crop yields and market prices and, thus, lowers risks in crop production [14]. Furthermore, temperate agroforestry can be more profitable than cropland monocultures [15], especially when negative externalities are considered [16]. Although farmers recognize and value the benefits of agroforestry, many are deterred by the increased labor and work complexity [17,18]. In this context, it was suggested that agroforestry practice would become more readily adopted by farmers if promoted and supported appropriately [17].

Apart from all the benefits, crop yields have been shown to decrease close to the trees as a result of resource competition [19,20,21,22]. While there is initial evidence that long-term yields in alley-cropping systems may be comparable to those in cropland monocultures [22], yield depressions in close proximity to the trees are inevitable and increase with tree height. Other than yield, crops cultivated in agroforestry systems are as healthy as crops cultivated in cropland monocultures [23].

Plant health and productivity as well as nutrient cycling are strongly driven by soil organisms, especially microorganisms, e.g., [24,25]. In recent decades, several soil organisms have been investigated in temperate agroforestry systems, ranging from soil macrofauna, e.g., [26], to microorganisms, e.g., [27]. While the influence of agroforestry systems on soil fauna and their functions has extensively been reviewed recently by Marsden et al. [28], the last review on soil microorganisms and their functions in agroforestry systems was published over one decade ago with little focus on temperate systems [29]. Since then, many new studies from temperate systems have been published, especially due to the rapid development of new molecular tools. In 2016, Banerjee and colleagues [30] published the first study that investigated soil bacterial communities in temperate agroforestry systems using 454 pyrosequencing. From 2019 onwards, several studies that used Illumina sequencing to profile microbial communities were published [31,32,33,34]. The work of Banerjee and colleagues [30] was also the first to quantify soil microbial groups in temperate agroforestry systems by using real-time PCR. As for amplicon sequencing, several studies that employed real-time PCR have been published since 2019, e.g., [35,36,37,38]. Additionally, various studies that used other non-molecular methods have been published in the last ten years, e.g., [27,39,40]. All these studies greatly enhanced our understanding of soil microbial communities and their functions in temperate agroforestry systems.

In this work, we review the available literature on the abundance, diversity, and function of soil microorganisms in alley-cropping agroforestry systems of the temperate zone. Based on our literature review, we provide recommendations for future studies of soil microorganisms in these systems.

## 2. Methods Used to Study Soil Microorganisms in Temperate Agroforestry Systems

Microorganisms inhabiting the soil of temperate alley-cropping agroforestry systems have been studied using a variety of methods ranging from microscopy techniques to modern high-throughput sequencing approaches. Among the most frequently applied methods are chloroform fumigation extraction (CFE) for the determination of the microbial biomass [27,35,36,39,40,41,42,43,44,45] and the measurement of potential enzyme activities [36,39,46,47,48,49,50,51]. Other popular methods to characterize soil microbial communities in temperate agroforestry systems include substrate-induced respiration [27,39,46,52] and phospholipid fatty acids (PLFA) analysis [27,40,50,53]. More recently, modern molecular techniques such as real-time PCR [30,32,33,35,36,37,38,54] and amplicon sequencing [30,31,32,33,34,37,55,56] have been applied to study the soil microbiome of temperate agroforestry systems. Since the late 1990s, these and other tools have generated a large body of literature regarding the abundance, diversity, and function of soil microbial communities in temperate alley-cropping agroforestry systems that we review in this article.

## 3. Trees in Agroforestry Systems Increase Soil Microbial Population Size

Soil microbial population sizes can be quantified using a variety of methods. While some techniques allow for the quantification of specific groups within the microbial population, other methods such as CFE estimate the overall microbial biomass by quantifying mainly C and N within microbial cells, termed microbial biomass C (MBC) and N (MBN), respectively. In 2019, it was demonstrated that poplar trees in alley-cropping systems increased MBC and MBN as compared to the neighboring crop rows [39]. The authors reported an increase from 52 to 81 in MBC and 31 to 63% in MBN in the tree rows as compared to multiple distances in the crop rows [39]. Three years later, Luo and coworkers verified these findings [45]. At two sites, the authors were able to show a gradual increase in MBC from the middle of the crop rows (365 mg kg^−1^) to the tree rows (572 mg kg^−1^) [45]. Similarly, MBN was 46 to 69% greater in the tree rows than in the middle of the crop rows [45]. These observations agree with findings from cherry-based intercropping systems [41]. Studies that investigated the microbial biomass in the tree rows, at multiple distances from the trees within the crop rows of the agroforestry systems, and in adjacent cropland monoculture systems found that MBC and MBN decrease with increasing distance from the tree rows [35,42]. Therefore, the positive effects of trees in agroforestry systems on the microbial biomass are not restricted to the tree rows and can extend gradually into the neighboring crop rows. The results of Lee and Jose [43] further indicate that the age of the agroforestry system affects the increase in microbial biomass. Within a 12-year-old maple-based agroforestry system with a maize—soybean rotation, however, no differences in MBC and MBN between the tree row and the crop row were observed [44].

In addition to the overall microbial population size, several studies quantified specific groups such as bacteria. Seiter et al. [57] were the first to quantify soil bacteria in temperate agroforestry systems. In their work, the authors studied the tree and crop rows of an alder tree—maize alley-cropping system compared to an adjacent maize monoculture and repeatedly determined the active and total bacterial biomass using fluorescein diacetate and fluorescein isothiocyanate staining, respectively. Their results showed that the tree row harbored more active bacteria than the crop row and the cropland monoculture, whereas the total bacterial biomass was not affected [57]. Quantification of soil bacteria using real-time PCR showed that tree rows in temperate agroforestry systems promote soil bacterial population size [30,32,35,37,54]. For example, copy numbers of bacterial 16S rRNA genes were 76 to 96% greater in the tree rows of poplar-based agroforestry systems than in neighboring cropland monocultures [54]. Furthermore, studies that sampled at different distances from the trees within the crop rows and included a cropland monoculture as a reference land use were able to show that bacterial abundance gradually decreased from the tree rows through the crop rows to the cropland monoculture [35,37,54]. Using PLFA, Guillot et al. [27] reported a greater abundance of gram-positive (GPB) and gram-negative bacteria (GNB) as well as Actinobacteria in the tree row of a walnut—pea alley-cropping than in the crop row as well as in an adjacent pea monoculture system. The authors were also able to demonstrate that the abundance of GPB, GNB, and Actinobacteria declined gradually with increased distance from the trees [27]. Likewise, Unger et al. [50] showed greater quantity of bacterial and GNB PLFAs in contour buffer strips planted with different oak species than in crop rows (maize–soybean rotation). In 2020, we quantified nine taxonomic groups of soil bacteria in three poplar-based temperate agroforestry systems using real-time PCR and showed that six out of nine groups of bacteria (Acidobacteria, Actinobacteria, Alpha- and Gammaproteobacteria, Firmicutes, and Verrucomicrobia) were promoted by the tree rows while three groups (Bacteriodetes, Betaproteobacteria, and Gemmatimonadetes) remained unaffected [54]. Similar to the study of Guillot et al. [27], the groups that were promoted by the tree rows showed a gradual decline in abundance with increasing distance from the trees [54].

The first investigation of soil fungi in temperate agroforestry systems was conducted by Seiter et al. [57]. By measuring FDA-stained hyphae under an epifluorescence microscope, the authors estimated the active fungal biomass and found greater active biomass of fungi in the tree row than in the crop row and a nearby cropland monoculture [57]. Similar results were obtained by Beuschel and coworkers [39]. They quantified ergosterol in soil to estimate the biomass of saprotrophic and ectomycorrhizal fungi and found 98 to 133% higher ergosterol levels in the tree rows than at multiple distances from the trees within the crop rows of agroforestry systems. Likewise, quantification of fungal 18S rRNA genes by real-time PCR revealed that tree rows increased soil fungal population size [35,37,54]. Furthermore, studies that measured fungal population size at different distances from the trees within the crop rows of agroforestry systems as well as adjacent cropland monoculture systems were able to show that fungal abundance decreases as the distance to the trees increases [27,35,37,54]. Basidiomycota have been shown to be particularly promoted by tree rows with increases of up to more than two orders of magnitude in the tree rows as compared to cropland monocultures [37,54].

The increase in the fungal population size through tree rows has not (yet) been detected in young agroforestry systems (less than five years of age) [33,36], and thus, appears to be limited to older systems. While a few studies showed no alteration of the fungi–to–bacteria ratio [27,54], several studies reported an increase in the ratio in the trees rows of agroforestry systems [35,38,39,50,57], suggesting that fungi benefit more from agroforestry practice than bacteria.

The by far most frequently investigated group of soil fungi in temperate agroforestry systems are arbuscular mycorrhizal fungi (AMF). Lacombe et al. [53] found an increase in AMF fatty acids in the crop rows of agroforestry systems as compared to cropland monoculture systems. Although Unger et al. [50] quantified PLFAs of AMF within an oak-based agroforestry system, the authors did not separate AMF from other types of mycorrhizae in their data analysis. The authors detected a greater abundance of mycorrhizal PLFAs in the tree row than in the crop row [50]. Bainard et al. [58], however, found no differences in AMF spore density, hyphal length, and root colonization between plots of cropland monocultures (corn, soybean, and winter barley) and adjacent plots within an agroforestry system containing *inter alia* white ash, silver maple, and Norway spruce. Within an alley-cropping system that combined rows of poplar, white ash, and black walnut trees with rows of soybean, Chifflot and coworkers [59] reported a greater abundance of AMF spores in the crop row than in the tree row. Therefore, the influence of agroforestry practice on the abundance of AMF communities remains uncertain.

Overall, soil microbial populations benefit from the presence of tree rows in temperate alley-cropping systems. Several studies also showed that the positive effect of tree rows in agroforestry systems on the microbial population size can extend gradually into the crop rows.

## 4. Do Agroforestry Systems Affect Soil Microbial Community Composition and Diversity?

The investigation into soil microbial diversity has improved tremendously through the use of molecular tools. Several recent papers have assessed the diversity of soil microbial communities in temperate agroforestry systems using tools such as amplicon sequencing, terminal restriction fragment length polymorphism (T-RFLP), and denaturing gradient gel electrophoresis (DGGE). Here, we compile the available literature on the alpha and beta diversity of soil microbial communities.

In 2013, Bardhan and colleagues [60] used DGGE to profile soil bacterial communities within a silver maple-based agroforestry system and found that the soil bacteria in the tree rows and the center of the crop rows did not differ in terms of alpha diversity and community composition. The limitations of DGGE to profile complex communities are well known, e.g., [61]. The authors detected a maximum of 28 DGGE bands within their DGGE profiles, which is only a fraction of what modern amplicon sequencing techniques such as Illumina sequencing can recover. Thus, modern amplicon sequencing techniques allow for a more in-depth analysis of soil microbial communities. Recent amplicon sequencing studies of soil bacterial communities with sufficient sequencing depth validate the findings of Bardhan et al. [60] by revealing that temperate agroforestry does not increase the alpha diversity of soil bacteria [30,32,34].

In contrast to alpha diversity, several studies showed that tree rows in temperate agroforestry systems alter the composition (beta diversity) of soil bacteria. For example, Banerjee et al. [30] reported that bacterial community composition differs between plots with trees and crops in Canadian alley-cropping systems. Likewise, Beule and Karlovsky [32] found altered community composition in the tree rows as compared to the crop rows of agroforestry systems and monoculture cropland systems. These findings also agree with the investigation of the microbial community composition using PLFA analysis [27,50,53]. A recent study that performed amplicon sequencing of bacterial 16S rRNA genes in poplar-based alley-cropping systems found that several bacterial genera were promoted by either tree rows or crop rows and cropland monocultures [32]. For example, the relative abundances of *Bradyrhizobium*, *Flavobacterium*, *Mesorhizobium*, and *Sporocytophaga* spp. were promoted by the tree rows while *Bacillus*, *Lysobacter*, *Nitrosospira*, and *Nitrospira* spp. were promoted by the crop rows and cropland monocultures [32]. At the same sites, similar patterns of relative abundances were reported for fungi. Tree rows increased the relative abundances of ectomycorrhizal genera such as *Cortinarius*, *Geopora*, and *Inocybe,* while crop rows and cropland monocultures favored affiliates of the genera *Alternaria*, *Fusarium*, *Microdochium,* and *Mortierella*.

While the Shannon diversity of soil fungi in agroforestry systems has been shown to moderately increase with increasing distance from the tree rows into the crop rows [34,37], species richness was found to decrease with increasing distance from the trees and was lowest in cropland monoculture systems [37]. The effects of agroforestry on community composition were more pronounced. Tree rows in temperate alley-cropping systems strongly shape soil fungal communities and the effects partly extend into the crop rows [37]. Furthermore, the integration of poplar tree rows in arable land has been shown to alter the community composition of soil fungi as soon as six months after agroforestry establishment [33], indicating a rapid adaption of soil fungi to the trees.

In 2020, van Tuinen et al. [56] used amplicon sequencing to inventory AMF communities in the roots of walnut trees and maize plants within a walnut—maize alley-cropping system and found distinct community composition patterns. Another investigation within two walnut—wheat alley-cropping systems sampled the roots of walnut trees, wheat plants, and various herbaceous plants in the tree rows as well as at multiple distances from the tree rows within the crop rows [31]. Similar to the observations of van Tuinen et al. [56], the authors reported differences in the community composition of AMF on the roots of the trees, crops, and herbaceous plants [31]. Additionally, greater alpha diversity of AMF was found in the crop rows as compared to the tree rows [31]. Using PCR-RFLP, Chifflot et al. [59] reported greater Shannon diversity of AMF in soybean roots as compared to poplar roots in a Canadian alley-cropping system. Another study using T-RFLP found an increased phylotype richness of AMF on maize roots sampled in an agroforestry system as compared to maize cultivated in a cropland monoculture system [58]. In addition to phylotype richness, the authors showed distinct AMF community compositions between the two systems (i.e., agroforestry vs. cropland monoculture) [58]. Lacombe et al. [53] used PLFA to compare the community composition of AMF in rows of soybean in two agroforestry systems with nearby soybean monocultures. At one of their study sites, they detected an increase in beta diversity within the soybean rows intercropped with poplar, white ash, and black walnut as compared to the soybean monoculture system [53].

Overall, soil microbial diversity in temperate alley-cropping agroforestry systems is greater than in cropland monoculture systems. This is mainly due to increased beta diversity within the agroforestry systems rather than an increase in alpha diversity: tree rows strongly shape soil microbial communities and harbor a microbiome that is compositionally distinct from that of the neighboring crops. In consequence, the introduction of a tree-row associated soil microbiome into arable land through agroforestry increases the overall diversity of the system.

## 5. Functions of Soil Microorganisms in Temperate Agroforestry Systems

Microbial activities in temperate agroforestry systems have frequently been investigated by measuring potential enzyme activities. Within poplar-based alley-cropping systems, the activities of β-glucosidase, β-xylosidase, N-acetyl-β-glucosaminidase, and tyrosine-aminopeptidase were greater in the tree rows than at multiple distances from the trees within the crop rows [39]. Likewise, studies that investigated enzyme activities within an agroforestry system in Missouri (USA) found increased FDA hydrolysis, β-glucosidase, and glucosaminidase enzyme activities in contour buffer strips with oak trees as compared to the crop rows [47,48,50]. For example, Udawatta and colleagues [47] reported an increase of 37 to 70% for the activity of these three enzymes in the tree rows as compared to the crop rows. Dehydrogenase enzyme activity was reported to not differ between the buffer strips and the crop rows [47,48,50]. In 2016, Weerasekara and coworkers [51] revisited the site and found no differences in FDA hydrolysis and β-glucosidase activities but higher activities of glucosaminidase and dehydrogenase in the buffer strip than in the crop rows. Two other studies carried out in Missouri (USA) investigated the same enzymatic activities at different soil depths (0–10 cm, 10–20 cm, and 20–30 cm) within maple-based alley-cropping systems [46,49]. While Mungai et al. [46] reported greater β-glucosidase activities in the tree row than in the crop row below 10 cm soil depth but lower FDA hydrolysis activity in the upper 10 cm topsoil, Udawatta et al. [49] reported no differences in FDA hydrolysis, β-glucosidase, glucosaminidase, and dehydrogenase activities. Recently, Clivot et al. [36] repeatedly determined six enzyme activities (arylsulfatase, β-glucosidase, leucine aminopeptidase, N-acetyl-β-glucosaminidase, phosphatase, and protease) in a young alfalfa—poplar alley-cropping system and found variable responses of the different enzyme activities across time. Overall, we observe a tendency towards increased potential enzyme activities in the tree rows of agroforestry systems as compared to the neighboring crop rows.

Measurements of microbial respiration in response to different substrates (substrate-induced respiration) or water (basal respiration) are common tools to investigate microbial functions. Using 31 C sources, Mungai and colleagues [46] detected greater substrate-induced respiration as well as a broader diversity of substrate utilization ability in the tree row than in the crop row of a maple-based intercropping system, indicating greater soil microbial functional diversity under the trees. In 2019, induced respiration of 16 substrates was tested in the tree rows as well as at three distances from the trees within the crop rows of three poplar-based alley-cropping systems [39]. The authors reported greater induced respiration rates of D-glucose (+44 to 59%), D-glucosamine (+39 to 53%), L-alanine (+76 to 95%), and protocatechuic acid (+45 to 62%) in the tree rows than in the crop rows [39]. In agreement with the findings of Mungai et al. [46], the authors also found increased diversity of substrate utilization ability in the tree rows as compared to the crop rows [39]. Although Guillot and coworkers [27] used just three substrates (glucose, trehalose, and alanine), the authors elegantly demonstrated that rows of hybrid walnut trees increase substrate-induced respiration by up to 142% as compared to the crop rows of the agroforestry system as well as to an adjacent cropland monoculture.

Few studies have quantified functional genes involved in soil-N cycling (N_2_ fixation, nitrification, and denitrification) in agroforestry systems using real-time PCR [32,38,45,54]. From 2020 to 2021, two studies showed that the abundance of the N_2_-fixation gene *nifH* was increased in the tree row as compared to the crop row and adjacent cropland monoculture at one of their three study sites with poplar-based alley-cropping systems. Therefore, the impact of rows of poplar trees on N_2_-fixing bacteria (such as cyanobacteria) remains yet to be thoroughly investigated [32,54]. So far, the impact of agroforestry on ammonia-oxidizing archaea (AOA) and bacteria (AOB) (involved in nitrification) has been investigated twice by quantifying ammonia monooxygenase subunit A (*amoA*) genes [38,54]. While the abundance of AOA remained rather unaffected by agroforestry, the abundance of AOB was suppressed by the tree rows, *cf.* [38,54]. In agreement with these findings, Beaudette et al. (2010) detected greater potential for nitrification with increasing distance from the trees within a poplar—oilseed rape alley-cropping system. The abundance of genes involved in denitrification (*napA*, *narG*, *nirK*, *nirS*, and *nosZ* clade I and II) was increased through agroforestry and was greatest in the tree rows [54]. Compared to adjacent cropland monoculture systems, tree rows in agroforestry systems increased the abundance of *napA*, *narG*, *nirK*, *nirS*, and *nosZ* clade I and II genes by up to 266, 179, 250, 196, 182, and 88%, respectively [54]. Although N-cycling gene abundances are often used to predict gaseous N fluxes (N_2_O in particular) from soil, it has to be noted that the quantification of functional genes reflects the genetic potential of a functional group and may not translate into actual soil functions under field conditions [45]. Field-based measurements of N_2_O emissions in temperate agroforestry systems are rare and inconclusive but show a tendency towards reduced N_2_O emissions in proximity to the tree rows *cf.* [62,63,64,65]. Recently, Luo et al. [45] demonstrated that temperate agroforestry systems can decrease gross N_2_O emissions by 6 to 36% and increase gross N_2_O uptake by 27 to 42%. Field measurements of other greenhouse gases were more consistent. For example, the few studies that examined CH_4_ fluxes under field conditions consistently showed that the integration of tree rows through agroforestry increased CH_4_ uptake [63,64,65] and has been shown to depend on the distance from the trees [65]. Rows of trees in temperate agroforestry systems have repeatedly been shown to increase soil CO_2_ emissions due to increased root respiration of the abundant tree root biomass as well as stimulated microbial respiration through increased C input by the trees, e.g., [12]. Although tree rows increase soil CO_2_ emissions, temperate agroforestry systems sequester larger amounts of C than cropland monocultures through the biomass of the trees as well as increased SOC stocks [12,66,67].

Overall, tree rows in temperate alley-cropping systems increase the catabolic potential, substrate-use efficiency, and the functional diversity of soil microorganisms as compared to crop rows and cropland monoculture systems. These effects are mostly limited to the tree rows and do not extend into the neighboring crop rows. Additionally, populations of functional microbial groups involved in N cycling have been shown to respond to the integration of trees in arable land. Population sizes of denitrifying microorganisms increased in tree rows as compared to crop rows and cropland monoculture systems. Conversely, tree rows decreased the population size of certain bacteria involved in nitrification (i.e., AOB). The integration of trees through agroforestry increases CO_2_ emissions (autotrophic and heterotrophic respiration, see above) and CH_4_ uptake, while the impact on N_2_O is less consistent across studies but showed a tendency towards lower N_2_O emissions in the tree rows than the crop rows and cropland monoculture systems. Furthermore, fluxes of CO_2_, CH_4_, and N_2_O have shown to be affected by the distance from the tree rows.

## 6. Potential Drivers of Microbial Abundance, Diversity, and Functions

After decades of agroforestry research in the temperate zone, it is widely recognized that the integration of trees in arable land through alley cropping improves soil quality (i.e., biological, chemical, and physical soil properties). Here, we list the main soil alterations through agroforestry and discuss how these changes can shape the soil microbiome with a focus on our findings above. We acknowledge though that these changes act simultaneously, thus, as in other complex systems, it is difficult to disentangle their individual impacts on the soil microbial community.

### 6.1. Different Soil Management

In contrast to the majority of cropland monoculture systems, tree rows in temperate agroforestry systems are not tilled. It is evident that distinct tillage regimes differently affect soil microbial communities, e.g., [68]. No-till systems generally increase microbial population size and activity as compared to tillage systems, e.g., [69]. Furthermore, differences in community composition (beta diversity) but not alpha diversity have been reported between conventional tillage and no-till systems [70]. Our review revealed increased microbial population size and activity as well as altered microbial community composition but unchanged alpha diversity in the tree rows (no-till) as compared to the crop rows and cropland monoculture systems (usually tilled). We suggest that differences in tillage regimes contribute to the observed patterns of microbial abundance, diversity, and functionality. Agroforestry studies with crop rows that are tilled or not tilled within the same system would be required to prove this assumption; however, such studies have not yet been conducted. Additionally, tree rows in temperate agroforestry systems typically do not receive fertilizer. Fertilization applications are well-known to affect the population size, composition, and function of the soil microbial communities, e.g., [71]. Thus, we expect that differences in fertilization within agroforestry systems accounted for some of the observed alterations of the soil microbial community. To specifically test for the effects of fertilization on soil microorganisms in temperate agroforestry systems, we suggest a study design in which fertilized and unfertilized crop rows are investigated simultaneously in the same agroforestry system. Overall, we see great potential for innovative study designs that aim to disentangle the effects of different management practices within agroforestry systems on soil microorganisms.

### 6.2. Different Plants and Cultivation Cycles

Tree rows in temperate alley-cropping agroforestry systems are comprised of woody perennial plants instead of annual crops. The length of the rotation cycle of the tree component is tree species dependent. The aboveground biomass of fast-growing trees with high biomass production (e.g., hybrid poplar trees) can be harvested repeatedly after few years, whereas trees that produce high-quality timber (e.g., walnut trees) are harvested only once after several decades. The increased cultivation cycle length of the trees as compared to the crops allows for the establishment of an herbaceous vegetation layer below the trees that enhances plant diversity [72]. In addition to its persistence and enhanced plant diversity, the above- and belowground biomass per area unit of the tree rows exceeds that of the crop rows by far. Therefore, different quantities and qualities of root exudates are expected in soil in the tree rows as compared to the crop rows. The influence of root exudate quantity and quality on soil microorganisms has been explored extensively, e.g., [73,74]. It can be expected that increased root exudate quantity and diversity in the tree rows contribute to the increased microbial population size and functional diversity as well as altered community composition in the tree rows as compared to the crop rows and cropland monoculture systems.

Aboveground tree litter in temperate agroforestry systems usually remains on site and decreases exponentially with increasing distance from the trees, e.g., [75]. Following the concept of nutrient capturing through tree roots and nutrient provisioning through leaf litter, e.g., [6], an increase in soil fertility can be expected in proximity to the trees. Recently, Pardon and colleagues [7] proved this assumption for poplar-based systems by showing that poplar tree rows in alley-cropping systems do not just increase soil fertility (SOC and available nutrient concentrations), but that this effect extends gradually into the crop rows. Soil fertility includes the biological, chemical, and physical fertility of soils and is closely interrelated with soil microorganisms. For example, microorganisms in soils are key drivers of soil–nutrient cycling (e.g., through decomposition of organic matter and nutrient release [76]) and can improve soil aggregation (e.g., through hyphal networks [77]). Conversely, edaphic soil properties strongly select for soil microbial communities. For example, the strong influence of soil pH on soil bacteria has been described over a century ago [78]. Therefore, the distribution of tree leaf litter and the resulting gradual increase in soil fertility likely contributed to the observed microbial abundance patterns within agroforestry systems (i.e., increased soil microbial abundance with increasing proximity to the trees).

## 7. Implications for Future Studies

Our literature review revealed that soil microorganisms in temperate alley-cropping agroforestry systems have been investigated applying a variety of spatiotemporal study designs. Here, key elements that we consider of importance when studying soil microorganisms in these systems are discussed.

### 7.1. Reference Land Use

We encourage researchers to include a reference land-use system without trees to enable a comparison between cropland agroforestry systems and the default agricultural land-use system: arable land. For temperate cropland alley-cropping agroforestry systems, this would require the inclusion of a spatially close arable cropland managed identically to the crop rows of the agroforestry system. Ideally, the initial soil conditions of the reference land-use system and the agroforestry system should be identical. Several previous studies highlighted the importance of such comparisons for the evaluation of agroforestry systems. We recognize the effort associated with including a reference land-use system; however, we argue that this will enable a fair comparison between agroforestry and other land-use systems.

### 7.2. Spatial Heterogeneity

We further believe that future studies should account for the spatial heterogeneity introduced by the trees by sampling along transects spanning from the tree rows to a reasonable distance within the crop rows. Such study designs will also enable area-based weighting of parameters as demonstrated by Schmidt et al. [79] who collected data at multiple distances from the tree rows within the crop rows of agroforestry systems and used area-weighted averages to represent the crop rows. Similarly, we used area-weighted averages of phytopathogen abundances and mycotoxin concentrations in crops cultivated in the crop rows of agroforestry systems to enable a direct comparison with crops grown in adjacent monoculture cropland systems [23]. Recently, Luo et al. [45] extended this by including the tree and crop rows in their area-weighted averages to represent agroforestry systems as a whole.

### 7.3. Soils Are Three-Dimensional Systems

Spatial heterogeneity within the agroforestry systems not just occurs horizontally but also vertically. For example, deep-rooting roots of trees present below the rooting zone of the neighboring crops can take up leached nutrients (‘safety-net role of tree roots’; [5]) and, thus, act as nutrient pumps [6]. A few studies have investigated microbial communities at different depths in agroforestry systems [39,44,46,49] and found that differences between the trees and crops may vanish with increasing soil depth *cf.* [39,46]. However, no investigations on soil microbial communities in temperate agroforestry systems below 30 cm soil depth exist. Exploring microbial communities across different depths, including subsoil, in agroforestry systems will greatly advance our understanding of the ecology and function of microorganisms in these systems and help to generate new research directions. For example, we speculate that tree-root associated microorganisms in subsoil contribute significantly to the safety-net role of the trees by increasing nutrient capturing and uptake by the roots. Figuratively speaking, we hypothesize that tree-root associated microorganisms decrease the mesh size of the safety net and, thus, support nutrient pumping. As before, however, we recognize that sampling multiple soil depths increases sampling efforts and may not always be feasible. Overall, so far, soil microbiologists have literally only just scratched the surface of agroforestry systems.

### 7.4. Microorganisms Are More than Just Bacteria and Fungi

Almost all studies that distinguished among different groups of soil microorganisms investigated bacteria and fungi, leaving other microorganisms understudied. For example, so far, no studies on the abundance (except for AOA [38,54]), community composition or diversity of archaea in temperate agroforestry systems exist. Likewise, information on the abundance of protozoa is scarce [50], and data on their community composition and diversity are lacking. Similarly, data on terrestrial microalgae are absent. Even key functional groups of bacteria and fungi involved in relevant ecosystem processes (e.g., N_2_-fixing bacteria) are either not studied or highly understudied. Therefore, we encourage researchers to go beyond bacteria and fungi and explore other relevant groups of soil microorganisms in temperate alley-cropping agroforestry systems.

### 7.5. Multiple Study Sites and Repeated Sampling

Discrepancies among study results are likely due to site-specific differences such as different tree and/or crop species, soil management, fertilization regimes, and ages of the systems. We agree with Beuschel et al. [39], who argued that these site-specific differences cannot be evaluated, since many studies investigated only a single site. To meet the diversity of temperate alley-cropping agroforestry systems, we recommend that future studies should investigate multiple sites whenever possible. Moreover, our review revealed that so far, the vast majority of studies were conducted in only five different countries, namely Canada, China, France, Germany, and the USA. Thus, future works should aim to include currently understudied regions to obtain a more complete picture of agroforestry systems in the temperate zone.

Since many agroforestry systems in the temperate zone integrate fast-growing trees with rotation times of around five years, the tree component has fast temporal dynamics. Similarly, crop rotations in the temperate zone commonly span around three to seven crops and may additionally include cover crops. Thus, the crop component of agroforestry systems is also dynamic in time. Finally, differences in soil management and fertilization are directly linked to the cultivated type of crop. Altogether, time is an important factor in agroforestry systems that should be considered when studying soil microorganisms. Although repeated sampling is rather common in other disciplines and often inevitable to understand soil processes, only few studies investigated soil microorganisms in temperate agroforestry systems across time. The investigation of multiple study sites with different tree species, ages, and rotation cycles as well as crop rotations (space–for–time substitution) would be a widely accepted solution to circumvent the issue of repeated sampling in agroforestry systems.

## 8. Conclusions

Our review revealed that tree rows in temperate alley-cropping agroforestry systems increase soil microbial population sizes, and that this beneficial effect can extend gradually into the crop rows. Additionally, fungi may benefit more than bacteria, as several studies indicated an increase in the fungi:bacteria ratio. Agroforestry systems increase soil microbial diversity mainly through the establishment of a tree–row association microbiome that is compositionally distinct from the microbiome of the crop rows (i.e., increased beta diversity within agroforestry systems) rather than increased alpha diversity. Furthermore, soil microbial populations in the tree rows of agroforestry system have greater catabolic potential and substrate-use efficiency and are functionally more diverse than those in the neighboring crop rows or in cropland monoculture systems. Microbial communities in soil of agroforestry systems are likely shaped by multiple factors (e.g., soil management, plant species, and cultivation cycles) that act simultaneously but to varying degrees across space and time. To disentangle the effects of these factors on soil microbial communities, future studies require study designs adapted to the complexity of these systems. Overall, temperate alley-cropping agroforestry systems increase soil microbial abundance, diversity, and functionality as compared to cropland monocultures, which is expected to contribute to enhanced biological soil fertility in these systems.

## Data Availability

Not applicable.
